# Theory of the Universal Medicine

**Published:** 1894-12-15

**Authors:** 


### The Panacea in English Medicine.

### I.—THEORY OF THE UNIVERSAL MEDICINE.

The term Panacea can be traced in medical vocabulary as far back as the history of medicine itself. Mythologically Panacea was the daughter of Esculapius and Hygeia, and devoted herself to the healing of mankind. It is interesting to trace the manner in which this word, signifying "all heal," came in the course of centuries to have two widely different meanings attached to it, and became, partly from this confusion of ideas, a rallying point round which some of the fiercest battles in medical history have been fought.

The term panacea was very early applied to medicines of any special value; but it was in this sense by no means intended to convey any notion of universal application, still less of unlimited powers of conferring health. Various herbs, highly esteemed among the ancients, gained the title of Panacea, such as the Panacea of Chiron or Nelianthemum vulgare; the Panacea of Hercules or Opoponax; the Panacea of Esculapius or ferula. Galen gave the name of Panacea to a favourite compound, of which the principal ingredient was horehound. The panacea, or elixir vitæ, of Paracelsus was mainly charged with aloes. In China the word is applied to the plant ginseng, which is sold for its weight in gold, and is believed to have very wonderful medical properties. But in all these instances, and many others where the kindred word catholicon is used, no omnipotent value is implied. Frequently a happy combination of various drugs, a plaster, electuary, or oil is thus described, such as the "emplastra epuloticon Andromachi"; or the celebrated catholicum electuary composed of rhubarb, liquorice, tamarinds, chicory, and many other ingredients.

But a far other value was assigned by certain students of medicine from very early times to the word Panacea. Deep reasoning on the nature and causes of disease and the source of life led them to a faith, seldom openly avowed, but most tenaciously preserved through life-long disappointment, in the existence somewhere in nature of a healing agency able to vanquish every form of decay and disease. This agency was the panacea. The discovery of this precious drug, equally efficacious in every illness, for every age and every person, was, according to the prevailing tone of the century, the cherished dream of divine, philosopher, physician or alchemist; of the most learned and scientific enquirer, or the most ignorant and reckless quack salver. The uncertainty of the quest lent it half its fascination. Researches extending over a life time and exhausting every resource of money, influence and learning, might prove abortive; yet the secret lay close at hand and a child might hap upon it. It is related of Hippocrates that he believed the panacea was to be found somewhere in the economy of nature, and even the learned Sydenham confessed to a lurking belief in its existence.

It is not, however, among the regular members of the medical profession that indications of a belief in a universal medicine are most likely to appear. The established traditions of medical science are so entirely at variance with the theory that any one drug can combat every symptom, and suit every constitution, that few enthusiasts could preserve such an irregular notion after going through the prescribed course of study. And, moreover, the stern discipline of facts and the daily demand for practical remedial agents are generally sufficient to keep the practitioner from the airy fabric of mystic speculation. It is among those who have arrived at medicine through bye-paths of independent meditation that the enthusiastic followers of the panacea are to be found. From its birthplace among the Arabians the belief spread in the course of centuries, under different strange disguises, through the whole of Europe. It was enforced with solemn argument, most weighty because most incomprehensible, by the more respectable of the Hermetic writers. It found a home in the crazy brain of the alchemist, and became for ever associated, somewhat unjustly, with the more crude delusion of the transmutation of metals. It grew at length so inextricably entangled in the meshes of occult observance and astrological imposture that its own followers became ashamed of it. As the more fanatical searchers after the secret, despairing of legitimate methods, plunged more deeply into the abominations of magic rites and devil worship, honest men lost first all hope of its discovery, then all faith in its existence. The conflict of opinion, slowly merging into definite denial of a possible panacea, may be traced in the medical works of the sixteenth and seventeenth centuries.

So ancient a belief, however, was but scotched, not killed. Again and again in medical history it reappears, dazzles the world for a brief period, and flashes away again into the darkness. The list of drugs which have been thought, at their first introduction, to merit the title is a long one, nor have drugs alone been exalted into this high position. Almost every agency in nature, from the stars to the modest microbe of modern science, has had its turn, and, perhaps, nothing is more captivating to the student of human nature than to observe the rapid conquest effected by the success of a favourite remedy over the mature judgment of knowledge and common sense.

---

The disinfection of railway carriages has recently been occupying the attention of the French Government, and the Minister of Health has addressed a letter to the railway companies asking their co-operation in advancing the safety of the public by disinfection of railway carriages in which sick patients have travelled. The letter is politely worded, and asks that officials should be on the look out for travellers, especially children, suffering from a hacking cough, oppressive respiration, and of pale appearance, such symptoms indicating diphtheria. As to the desirability of strict isolation of patients travelling with any infectious disease there can be no two opinions, but whether railway officials can be expected to diagnose a particular malady is another matter. It would be more reassuring were each train to carry its own doctor, and if each passenger were supplied with a certificate of health to be shown on demand.

